# Optimization and Implementation of Scaling-Free CORDIC-Based Direct Digital Frequency Synthesizer for Body Care Area Network Systems

**DOI:** 10.1155/2012/651564

**Published:** 2012-11-05

**Authors:** Ying-Shen Juang, Lu-Ting Ko, Jwu-E. Chen, Tze-Yun Sung, Hsi-Chin Hsin

**Affiliations:** ^1^Department of Business Administration, Chung Hua University, Hsinchu City 300-12, Taiwan; ^2^Department of Electrical Engineering, National Central University, Chungli City 320-01, Taiwan; ^3^Department of Microelectronics Engineering, Chung Hua University, Hsinchu City 300-12, Taiwan; ^4^Department of Computer Science and Information Engineering, National United University, Miaoli 360-03, Taiwan

## Abstract

Coordinate rotation digital computer (CORDIC) is an efficient algorithm for computations of trigonometric functions. Scaling-free-CORDIC is one of the famous CORDIC implementations with advantages of speed and area. In this paper, a novel direct digital frequency synthesizer (DDFS) based on scaling-free CORDIC is presented. The proposed multiplier-less architecture with small ROM and pipeline data path has advantages of high data rate, high precision, high performance, and less hardware cost. The design procedure with performance and hardware analysis for optimization has also been given. It is verified by Matlab simulations and then implemented with field programmable gate array (FPGA) by Verilog. The spurious-free dynamic range (SFDR) is over 86.85 dBc, and the signal-to-noise ratio (SNR) is more than 81.12 dB. The scaling-free CORDIC-based architecture is suitable for VLSI implementations for the DDFS applications in terms of hardware cost, power consumption, SNR, and SFDR. The proposed DDFS is very suitable for medical instruments and body care area network systems.

## 1. Introduction

**Figure 1:**
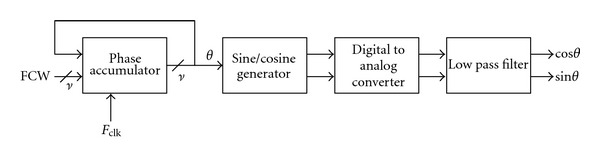
The conventional DDFS architecture.

Direct digital frequency synthesizer (DDFS) has been widely used in the modern communication systems. DDFS is preferable to the classical phase-locked-loop- (PLL-) based synthesizer in terms of switching speed, frequency resolution, and phase noise, which are beneficial to the high-performance communication systems.  Figure 1 depicts the conventional DDFS architecture [1], which consists of a phase accumulator, a sine/cosine generator, a digital-to-analog converter (DAC), and a low-pass filter (LPF). As noted, two inputs: the reference clock and the frequency control word (FCW) are used; the phase accumulator integrates FCW to produce an angle in the interval of [0,2*π*), and the sine/cosine generator computes the sinusoidal values. In practice, the sine/cosine generator is implemented digitally, and thus followed by digital-to-analog conversion and low-pass filtering for analogue outputs. Such systems can be applied in many fields, especially in industrial, biological, and medical applications [2–4].

The simplest way to implement the sine/cosine generator is to use ROM lookup table (LUT). However, a large ROM is needed [5]. Several efficient compression techniques have been proposed to reduce the lookup table size [5–10]. The quadrant compression technique can compress the lookup table and then reduce the ROM size by 75% [6]. The Sunderland architecture splits the ROM into two smaller memories [7], and the Nicholas architecture improves the Sunderland architecture to achieve a higher ROM-compression ratio (32 : 1) [8]. The ROM size can be further reduced by using the polynomial approximations [11–18] or CORDIC algorithm [19–27]. In the polynomial approximations-based DDFSs, the interval of [0, *π*/4] is divided into subintervals, and sine/cosine functions are evaluated in each subinterval. The polynomial approximations-based DDFS requires a ROM to store the coefficients of the polynomials and the polynomial evaluation hardware with multipliers. In the circular mode of CORDIC, which is an iterative algorithm to compute sine/cosine functions, an initial vector is rotated with a predetermined sequence of subangles such that the summation of the rotations approaches the desired angle [28, 29]. CORDIC has been widely used for the sine/cosine generator of DDFS [19–27]. Compared to the lookup table-based DDFS, the CORDIC-based DDFS has the advantage of avoiding the exponential growth of hardware complexity while the output word size increases [30–33].

In Figure 1, the word length of the phase accumulator is *v* bits; thus, the period of the output signal is as follows:

(1) $$\begin{array}{c} T_{o}=\frac{2^{v}T_{s}}{\text{FCW}}, \end{array}$$

where FCW is the phase increment and *T*_*s*_ denotes the sampling period. It is noted that the output frequency can be written by

(2) $$\begin{array}{c} F_{o}=\frac{1}{T_{0}}=\frac{F_{s}}{2^{v}}\cdot \text{FCW}. \end{array}$$

According to the equation above, the minimum change of output frequency is given by

(3) $$\begin{array}{c} \Delta F_{o,\min}=\frac{F_{s}}{2^{v}}\left(\text{FCW}+1\right)-\frac{F_{s}}{2^{v}}\text{FCW}=\frac{F_{s}}{2^{v}}. \end{array}$$

Thus, the frequency resolution of DDFS is dependent on the word length of the phase accumulator as follows:

(4) $$\begin{array}{c} \Delta F_{o}\geq \frac{F_{s}}{2^{v}}. \end{array}$$

The bandwidth of DDFS is defined as the difference between the highest and the lowest output frequencies. The highest frequency is determined by either the maximum clock rate or the speed of logic circuitries; the lowest frequency is dependent on FCW. Spurious-free dynamic range (SFDR) is defined as the ratio of the amplitude of the desired frequency component to that of the largest undesired one at the output of DDFS, which is often represented in dB_c_ as follows:

(5) $$\begin{array}{c} \text{SFDR}=20\log\left(\frac{A_{p}}{A_{s}}\right)=20\log\left(A_{p}\right)-20\log\left(A_{s}\right), \end{array}$$

where *A*_*p*_ is the amplitude of the desired frequency component and *A*_*s*_ is the amplitude of the largest undesired one.

In this paper, a novel DDFS architecture based on the scaling-free CORDIC algorithm [34] with ROM mapping is presented. The rest of the paper is organized as follows. In Section 2, CORDIC is reviewed briefly. In Section 3, the proposed DDFS architecture is presented. In Section 4, the hardware implementation of DDFS is given. Conclusion can be found in Section 5.

## 2. The CORDIC Algorithm

CORDIC is an efficient algorithm that evaluates various elementary functions including sine and cosine functions. As hardware implementation might only require simple adders and shifters, CORDIC has been widely used in the high speed applications.

### 2.1. The CORDIC Algorithm in the Circular Coordinate System

A rotation of angle *θ* in the circular coordinate system can be obtained by performing a sequence of micro-rotations in the iterative manner. Specifically, a vector can be successively rotated by the use of a sequence of pre-determined step-angles: *α*(*i*) = tan^−1^(2^−*i*^). This methodology can be applied to generate various elementary functions, in which only simple adders and shifters are required. The conventional CORDIC algorithm in the circular coordinate system is as follows [28, 29]:

(6) $$\begin{array}{c} x\left(i+1\right)=x\left(i\right)-\sigma \left(i\right)2^{-i}y\left(i\right), \end{array}$$

(7) $$\begin{array}{c} y\left(i+1\right)=y\left(i\right)+\sigma \left(i\right)2^{-j}x\left(i\right), \end{array}$$

(8) $$\begin{array}{c} z\left(i+1\right)=z\left(i\right)-\sigma \left(i\right)\alpha \left(i\right), \end{array}$$

(9) $$\begin{array}{c} \alpha \left(i\right)=\tan^{-1}2^{-i}, \end{array}$$

where *σ*(*i*) ∈ {−1, +1} denotes the direction of the *i*th micro-rotation, *σ*_*i*_ = sign⁡(*z*(*i*)) with *z*(*i*) → 0 in the vector rotation mode [34], *σ*_*i*_ = −sign⁡(*x*(*i*)) · sign⁡(*y*(*i*)) with *y*(*i*) → 0 in the angle accumulated mode [34], the corresponding scale factor *k*(*i*) is equal to $\sqrt{1+\sigma^{2}(i)2^{-2i}}$, and *i* = 0,1,…., *n* − 1. The product of the scale factors after *n* micro-rotations is given by

(10) $$\begin{array}{c} K_{1}=\prod_{i=0}^{n-1}k\left(i\right)=\prod_{i=0}^{n-1}\sqrt{1+2^{-2i}}. \end{array}$$

In the vector rotation mode, sin⁡ *θ* and cos⁡ *θ* can be obtained with the initial value: (*x*(0), *y*(0)) =  (1/*K*_1_, 0). More specifically, *x*_out_ and *y*_out_ are computed from the initial value: (*x*_in_, *y*_in_) = (*x*(0), *y*(0)) as follows:

(11) $$\begin{array}{c} \left[\begin{array}{c} x_{\text{out}}\\ y_{\text{out}} \end{array}\right]=K_{1}\left[\begin{array}{cc} \cos\theta & -\sin\theta \\ \sin\theta & \cos\theta \end{array}\right]\left[\begin{array}{c} x_{\text{in}}\\ y_{\text{in}} \end{array}\right]. \end{array}$$

### 2.2. Scaling-Free CORDIC Algorithm in the Circular Coordinate System

Based on the following approximations of sine and cosine functions:

(12) $$\begin{array}{c} \sin\alpha \left(i\right)≅\alpha \left(i\right)=2^{-i},\\ \cos\alpha \left(i\right)≅1-\frac{\alpha^{2}\left(i\right)}{2}=1-2^{-(2i+1)}, \end{array}$$

the scaling-free CORDIC algorithm is thus obtained by using (6), (7), and the above. In which, the iterative rotation is as follows:

(13) $$\begin{array}{c} \left[\begin{array}{c} x\left(i+1\right)\\ y\left(i+1\right) \end{array}\right]=\left[\begin{array}{cc} 1-2^{-(2i+1)} & 2^{-i}\\ -2^{-i} & 1-2^{-(2i+1)} \end{array}\right]\left[\begin{array}{c} x\left(i\right)\\ y\left(i\right) \end{array}\right],\\ z\left(i+1\right)=z\left(i\right)-2^{-i}. \end{array}$$

For the word length of *w* bits, it is noted that the implementation of scaling-free CORDIC algorithm utilizes four shifters and four adders for each micro-rotation in the first *w*/2-microrotations; it reduces two shifters and two adders for each microrotation in the last *w*/2-micro-rotations [24, 34, 35].

## 3. Design and Optimization of the Scaling-Free CORDIC-Based DDFS Architecture

In this section, the architecture together with performance analysis of the proposed DDFS is presented. It is a combination of the scaling-free-CORDIC algorithm and LUT; this hybrid approach takes advantage of both CORDIC and LUT to achieve high precision and high data rate, respectively. The proposed DDFS architecture consists of phase accumulator, radian converter, sine/cosine generator, and output stage.

### 3.1. Phase Accumulator

**Figure 2:**
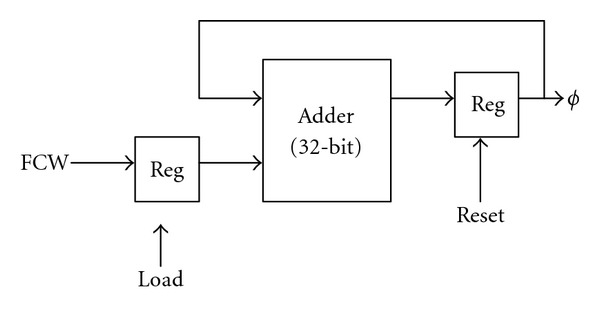
The phase accumulator in DDFS.

Figure 2 shows the phase accumulator, which consists of a 32-bit adder to accumulate the phase angle by FCW recursively. At time *n*, the output of phase accumulator is *ϕ* = (*n* · FCW)/2^32^ and the sine/cosine generator produces sin⁡((*n* · FCW)/2^32^) and cos⁡((*n* · FCW)/2^32^). The *load* control signal is used for FCW to be loaded into the register, and the *reset* signal is to initialize the content of the phase accumulator to zero.

### 3.2. Radian Converter

**Figure 3:**
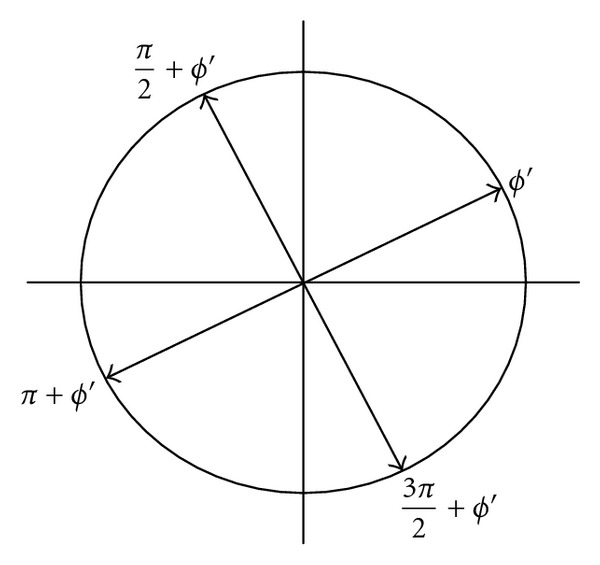
Symmetry-based map of an angle in either the second, third, or fourth quadrant to the corresponding angle in the first quadrant.

**Figure 4:**
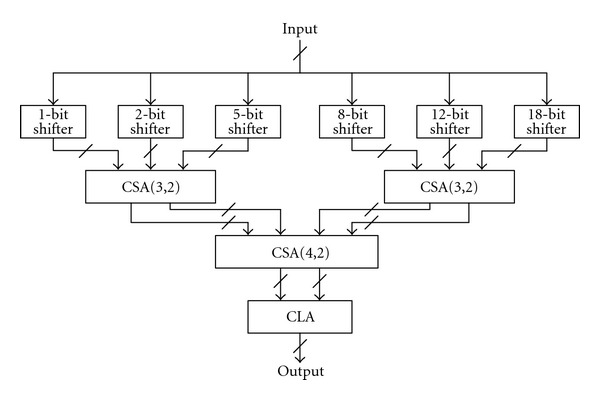
The constant (*π*/4) multiplier.

**Figure 5:**
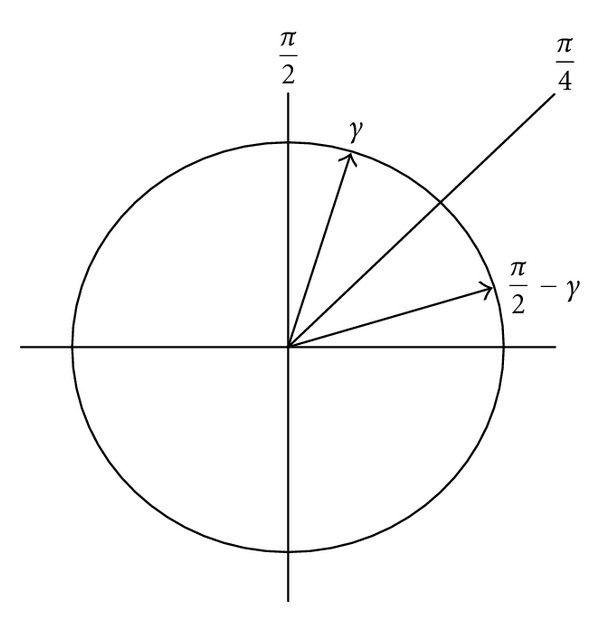
*π*/4-mirror map of an angle *γ* above *π*/4 to the corresponding angle *π*/2 − *γ* below *π*/4.

In order to convert the output of the phase accumulator into its binary representation in radians, the following strategy has been adopted. Specifically, an efficient ROM reduction scheme based on the symmetry property of sinusoidal wave can be obtained by simple logic operations to reconstruct the sinusoidal wave from its first quadrant part only. In which, the first two MSBs of an angle indicate the quadrant of the angle in the circular coordinate and the third MSB indicates the half portion of the quadrant; thus, the first three MSBs of an angle are used to control the interchange/negation operation in the output stage. As shown in Figure 3, the corresponding angles of *ϕ*′ in the second, third, and fourth quadrants can be mapped into the first quadrant by setting the first two MSBs to zero. The radian of *ϕ*′ is therefore obtained by *θ* = (*π*/4)*ϕ*′, which can be implemented by using simple shifters and adders array shown in Figure 4. Note that the third MSB of any radian value in the upper half of a quadrant is 1, and the sine/cosine of an angle *γ* in the upper half of a quadrant can be obtained from the corresponding angle in the lower half as shown in Figure 5. More specifically, as cos⁡*γ* = sin⁡((*π*/2) − *γ*) and sin⁡*γ* = cos⁡((*π*/2) − *γ*), the normalized angle can be obtained by replacing *θ* with *θ*′ = 0.5 − *θ* while the third MSB is 1. In case the third MSB is 0, there is no need to perform the replacement as *θ*′ = *θ*.

### 3.3. Sine/Cosine Generator

As the core of the DDFS architecture, the sine/cosine generator produces sinusoidal waves based on the output of the radian converter. Without loss of generality, let the output resolution be of 16 bits, for the sine/cosine generator consisting of a cascade of *w* processors, each of which performs the sub-rotation by a fixed angle of 2^−*i*^ radian as follows:

(14) $$\begin{array}{c} x\left(i+1\right)=\left(1-\sigma \left(i\right)2^{-(2i+1)}\right)x\left(i\right)+\sigma \left(i\right)2^{-i}y\left(i\right),\\ y\left(i+1\right)=\left(1-\sigma \left(i\right)2^{-(2i+1)}\right)y\left(i\right)-\sigma \left(i\right)2^{-i}x\left(i\right). \end{array}$$

**Figure 6:**
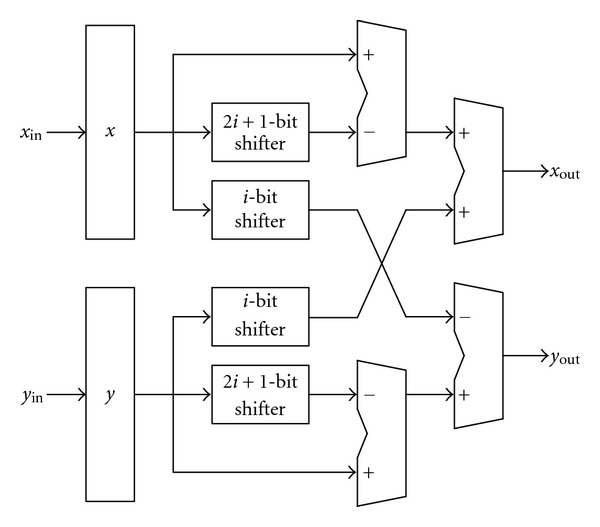
The CORDIC processor-A.

**Figure 7:**
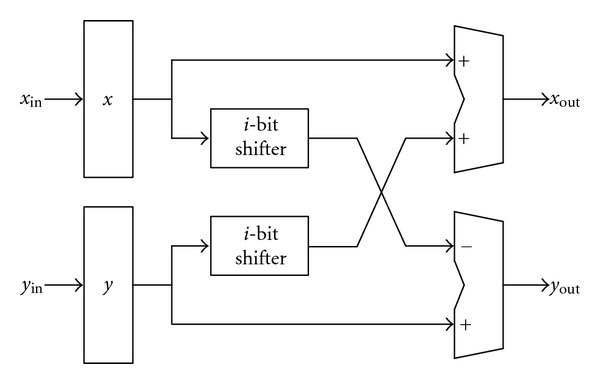
The CORDIC processor-B.

For 8 ≤ *i* < 16

(15) $$\begin{array}{c} x\left(i+1\right)=x\left(i\right)+\sigma \left(i\right)2^{-i}y\left(i\right),\\ y\left(i+1\right)=y\left(i\right)-\sigma \left(i\right)2^{-i}x\left(i\right), \end{array}$$

where *σ*(*i*) ∈ {1,0} representing the positive or zero subrotation, respectively.  Figure 6 depicts the CORDIC processor-A for the first 7 microrotations, which consists of four 16-bit adders and four 16-bit shifters. The CORDIC processor-B with two 16-bit adders and two 16-bit shifters for the last 9 microrotations is shown in Figure 7.

**Figure 8:**
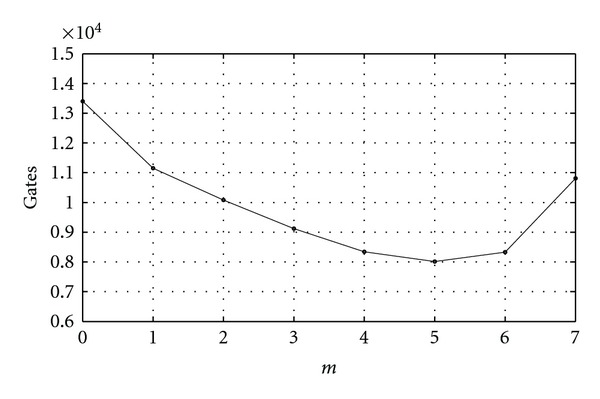
Hardware requirements with respect to the replaced CORDIC stages.

**Figure 9:**
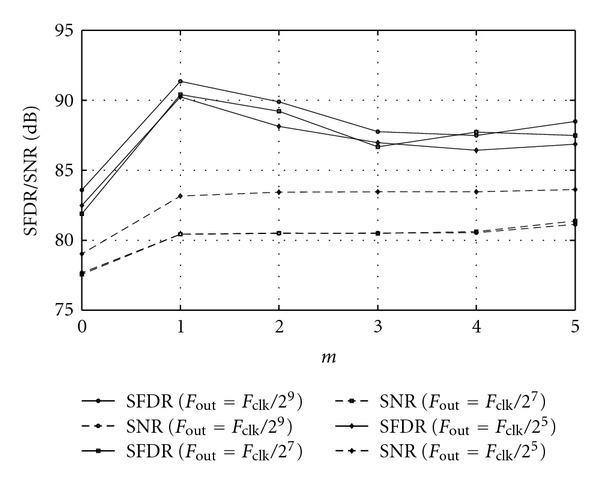
SFDR/SNRs with respect to the replaced CORDIC-stages.

The first *m* CORDIC stages can be replaced by simple LUT to reduce the data path at the cost of hardware complexity increasing exponentially.  Table 1 depicts the hardware costs in 16-bit DDFS with respect to the number of the replaced CORDIC-stages, where each 16-bit adder, 16-bit shifter, and 1-bit memory require 200 gates, 90 gates, and 1 gate [36], respectively.  Figure 8 shows the hardware requirements with respect to the number of the replaced CORDIC-stages [24].  Figure 9 shows the SFDR/SNRs with respect to the replaced CORDIC-stages [25]. As one can expect, based on the above figures, there is a tradeoff between hardware complexity and performance in the design of DDFS.

### 3.4. Output Stage

**Figure 10:**
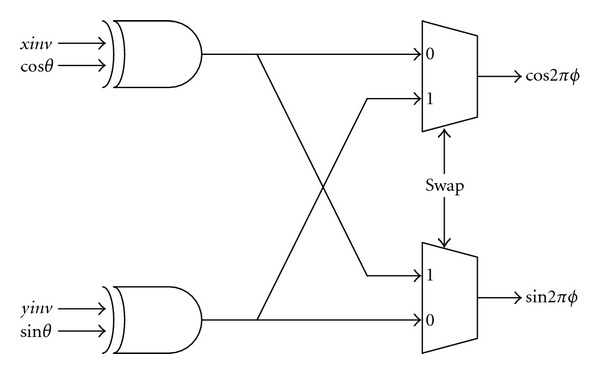
The output stage.

Figure 10 shows the architecture of output stage, which maps the computed sin⁡*θ* and cos⁡*θ* to the desired sin⁡*ϕ* and cos⁡*ϕ*. As mentioned previously, the above mapping can be accomplished by simple negation and/or interchange operations. The three control signals: *xinv*, *yinv*, and *swap* derived from the first three MSBs of *ϕ* are shown in Table 2. *xinv* and *yinv* are for the negation operation of the output and *swap* for the interchange operation.

## 4. Hardware Implementation of the Scaling-Free CORDIC-Based DDFS

**Figure 11:**
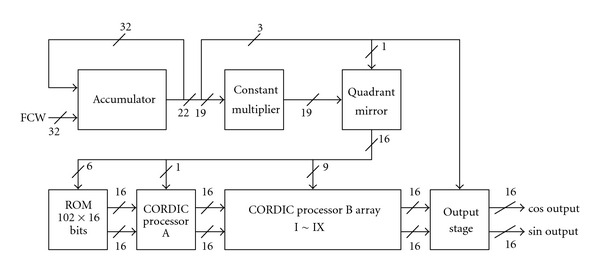
The proposed DDFS architecture.

**Figure 12:**
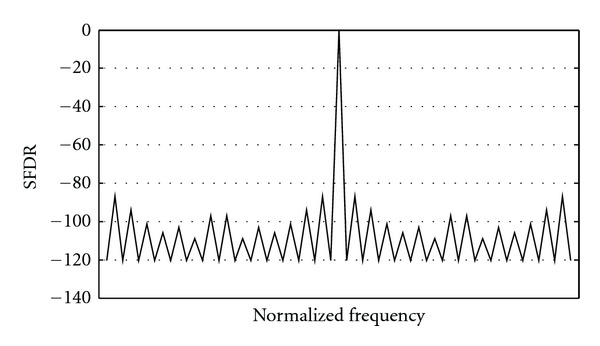
SFDR of the proposed DDFS architecture at output frequency *F*_clk_/2^5^.

In this section, the proposed low-power and high-performance DDFS architecture (*m* = 5) is presented.  Figure 11 depicts the system block diagram; SFDR of the proposed DDFS architecture at output frequency *F*_clk_/2^5^ is shown in Figure 12. As one can see, the SFDR of the proposed architecture is more than 86.85 dBc.

The platform for architecture development and verification has also been designed as well as implemented to evaluate the development cost [37–40]. The proposed DDFS architecture has been implemented on the Xilinx FPGA emulation board [41]. The Xilinx Spartan-3 FPGA has been integrated with the microcontroller (MCU) and *I*/*O* interface circuit (USB 2.0) to form the architecture development and verification platform. 

**Figure 13:**
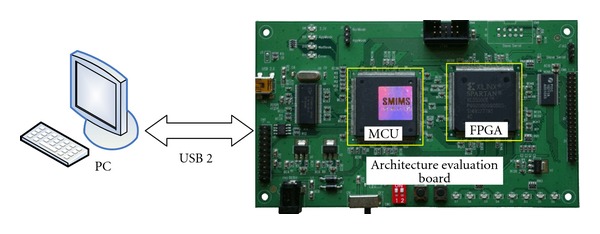
Block diagram and circuit board of the architecture development and verification platform.

Figure 13 depicts block diagram and circuit board of the architecture development and evaluation platform. In which, the microcontroller read data and commands from PC and writes the results back to PC via USB 2.0 bus; the Xilinx Spartan-3 FPGA implements the proposed DDFS architecture. The hardware code in Verilog runs on PC with the ModelSim simulation tool [42] and Xilinx ISE smart compiler [43]. It is noted that the throughput can be improved by using the proposed architecture, while the computation accuracy is the same as that obtained by using the conventional one with the same word length. Thus, the proposed DDFS architecture is able to improve the power consumption and computation speed significantly. Moreover, all the control signals are internally generated on-chip. The proposed DDFS provides both high performance and less hardware.

**Figure 14:**
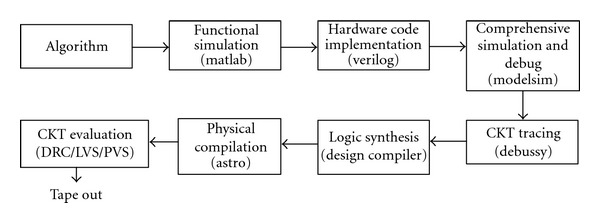
Cell-based design flow.

The chip has been synthesized by using the TSMC 0.18 *μ*m 1P6M CMOS cell libraries [44]. The physical circuit has been synthesized by the Astro tool. The circuit has been evaluated by DRC, LVS, and PVS [45].  Figure 14 shows the cell-based design flow. 

**Figure 15:**
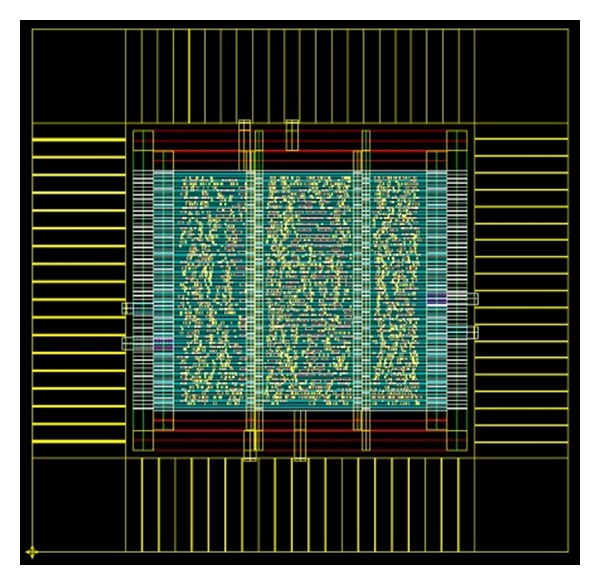
Layout view of the proposed scaling-free-CORDIC-based DDFS.

Figure 15 shows layout view of the proposed scaling-free CORDIC-based DDFS. The core size obtained by the Synopsys design analyzer is 452 × 452 *μ*m^2^. The power consumption obtained by the PrimePower is 0.302 mW with clock rate of 500 MHz at 1.8 V. The tuning latency is 11 clock cycles. All of the control signals are internally generated on-chip. The chip provides both high throughput and low gate count.

## 5. Conclusion

In this paper, we present a novel DDFS architecture-based on the scaling-free CORDIC algorithm with small ROM and pipeline data path. Circuit emulation shows that the proposed high performance architecture has the advantages of high precision, high data rate, and simple hardware. For 16-bit DDFS, the SFDR of the proposed architecture is more than 86.85 dBc. As shown in Table 3, the proposed DDFS is superior to the previous works in terms of SFDR, SNR, output resolution, and tuning latency [6, 17, 18, 26, 27]. According to the high performance of the proposed DDFS, it is very suited for medical instruments and body care network systems [46–49]. The proposed DDFS with the use of the portable Verilog is a reusable IP, which can be implemented in various processes with tradeoffs of performance, area, and power consumption.

## Figures and Tables

**Table 1:** The hardware costs in 16-bit DDFS with respect to the number of the replaced CORDIC stages (*m*: the number of the replaced CORDIC stages, 16-bit adder: 200 gates, 16-bit shift: 90 gates, and 1-bit ROM: 1 gate).

*m*	0	1	2	3	4	5	6	7
CORDIC processor requirement:								
CORDIC processor-A	7	5	4	3	2	1	0	0
CORDIC processor-B	9	9	9	9	9	9	9	8

Hardware cost:								
16-bit Adders	46	38	34	30	26	22	18	16
16-bit Shifters	46	38	34	30	26	22	18	16
ROM size (bits)	4 × 16	8 × 16	14 × 16	26 × 16	50 × 16	102 × 16	194 × 16	386 × 16

Total gate counts	13404	11148	10084	9116	8340	8012	8324	10816

**Table 2:** Control signals of the output stage.

MSB's of *ϕ*	*ϕ*	*xi* *nv*	*yi* *nv*	*sw* *ap*	cos⁡2*πϕ*	sin⁡⁡2*πϕ*
000	$0<2\pi \phi <\frac{\pi }{4}$	0	0	0	cos⁡*θ*	sin⁡⁡*θ*
001	$\frac{\pi }{4}<2\pi \phi <\frac{\pi }{2}$	0	0	1	sin⁡⁡*θ*	cos⁡*θ*
010	$\frac{\pi }{2}<2\pi \phi <\frac{3\pi }{4}$	0	1	1	−sin⁡*θ*	cos⁡*θ*
011	$\frac{3\pi }{4}<2\pi \phi <\pi$	1	0	0	−cos⁡⁡*θ*	sin⁡⁡*θ*
100	$-\pi <2\pi \phi <-\frac{3\pi }{4}$	1	1	0	−cos⁡⁡*θ*	−sin⁡⁡*θ*
101	$-\frac{3\pi }{4}<2\pi \phi <-\frac{\pi }{2}$	1	1	1	−sin⁡*θ*	−cos⁡*θ*
110	$-\frac{\pi }{2}<2\pi \phi <-\frac{\pi }{4}$	1	0	1	sin⁡⁡*θ*	−cos⁡*θ*
111	$-\frac{\pi }{4}<2\pi \phi <0$	0	1	0	cos *θ*	−sin⁡⁡*θ*

**Table 3:** Comparisons of the proposed DDFS with other related works.

DDFS	Kang and Swartzlander, 2006 [23]	Sharma et al., 2009 [26]	Jafari et al., 2005 [17]	Ashrafi and Adhami, 2007 [18]	Yi et al., 2006 [6]	De Caro et al., 2009 [27]	This work, Juang et al., 2012
Process (*μ*m)	0.13	—	0.5	0.35	0.35	0.25	0.18
Core area (mm^2^)	0.35	—	—	—	—	0.51	0.204
Maximum sampling rate (MHz)	1018	230	106	210	100	385	500
Power consumption (mW)	0.343	—	—	112	0.81	0.4	0.302
SFDR (dB_c_)	90	54	—	72.2	80	90	86.85
SNR (dB)	—	—	—	67	—	70	81.12
Output resolution (bit)	17	10	14	12	16	13	16
Tuning latency (clock)	—	—	33	—	—	—	11
